# Epigenetic drugs in cancer therapy

**DOI:** 10.1007/s10555-025-10253-7

**Published:** 2025-02-26

**Authors:** Amila Suraweera, Kenneth J. O’Byrne, Derek J. Richard

**Affiliations:** 1 School of Biomedical Sciences, Centre for Genomics and Personalised Health, Queensland University of Technology (QUT), 60 Musk Avenue, Kelvin Grove, QLD 4059 Australia; 2 Princess Alexandra Hospital, 199 Ipswich Road, Woolloongabba, QLD 4102 Australia

**Keywords:** Epigenetic drugs, Cancer therapy, DNA methyltransferase inhibitors, Histone deacetylase inhibitors, Histone methyltransferase inhibitors

## Abstract

Genetic and epigenetic modifications of DNA are involved in cancer initiation and progression. Epigenetic modifications change chromatin structure and DNA accessibility and thus affect DNA replication, DNA repair and transcription. Epigenetic modifications are reversible and include DNA methylation, histone acetylation and histone methylation. DNA methylation is catalysed by DNA methyltransferases, histone acetylation and deacetylation are catalysed by histone acetylases and deacetylases, while histone methylation is catalysed by histone methyltransferases. Epigenetic modifications are dysregulated in several cancers, making them cancer therapeutic targets. Epigenetic drugs (epi-drugs) which are inhibitors of epigenetic modifications and include DNA methyltransferase inhibitors (DNMTi), histone deacetylase inhibitors (HDACi), histone methyltransferase inhibitors (HMTi) and bromodomain and extra-terminal motif protein inhibitors (BETi), have demonstrated clinical success as anti-cancer agents. Furthermore, the combination of epi-drugs with standard chemotherapeutic agents has demonstrated promising anti-cancer effects in pre-clinical and clinical settings. In this review, we discuss the role of epi-drugs in cancer therapy and explore their current and future use in combination with other anti-cancer agents used in the clinic. We further highlight the side effects and limitations of epi-drugs. We additionally discuss novel delivery methods and novel tumour epigenetic biomarkers for the screening, diagnosis and development of personalised cancer treatments, in order to reduce off-target toxicity and improve the specificity and anti-tumour efficacy of epi-drugs.

## Introduction

The term epigenetics was first coined in 1942 by Conrad Waddington to establish a connection between the genotype and phenotype [1, 2]. Today, the term epigenetics refers to the study of reversible heritable changes in gene expression that are not due to alterations in the DNA sequence [3, 4]. Epigenetic modifications change DNA accessibility and thereby affect transcription, DNA replication and DNA repair. The main epigenetic mechanisms include histone modifications, DNA methylation, chromatin remodelling and non-coding RNA [3, 5, 6]. Histones are central components of chromatin which can be covalently modified. Histone N-terminal post-translational modifications include methylation, acetylation, phosphorylation, ubiquitination, sumoylation, ADP ribosylation, citrullination, proline isomerization, beta-N-acetylglucosamine and lactylation [7, 8]. The two most abundant and widely studied histone post-translational modifications include acetyl and methyl groups. While acetylated histones are less compact and facilitate transcription, methylated histones can be activating or repressive. DNA methyltransferases (DNMT) catalyse DNA methylation, histone acetyltransferases (HAT) add acetyl groups to histones, histone deacetylases (HDAC) remove acetyl groups, histone methyltransferases (HMT) catalyse histone methylation and histone demethylases catalyse the removal of methyl groups from arginine and lysine residues on histone tails. Enzymes that add methyl or acetyl groups to DNA or histones are known as ‘writers’, those that remove histone marks are known as ‘erasers’ and proteins that recognise DNA and histone modifications are known as ‘readers’ [7, 9–11].

**Fig. 1:**
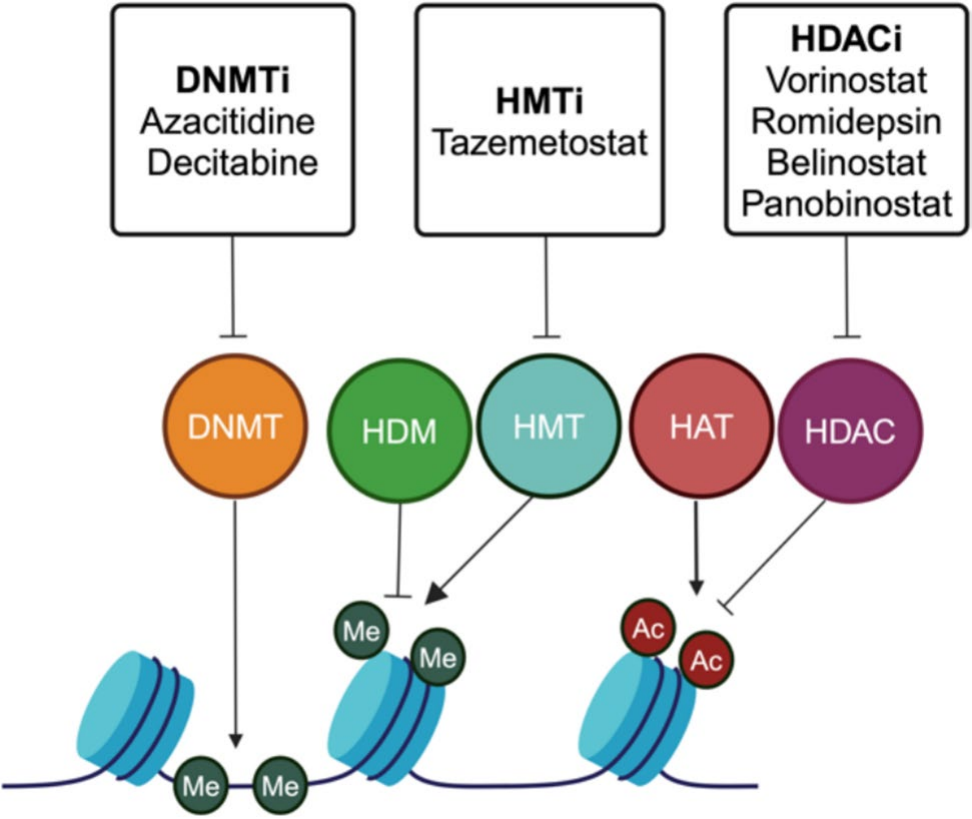
A schematic illustration depicting the role of DNA methyltransferases (DNMT), histone demethylases (HDM), histone methyltransferase (HMT), histone acetyltransferases (HAT) and histone deacetylases (HDAC), and names and mechanism of action of FDA approved DNA methyltransferase inhibitors (DNMTi), histone methyltransferase inhibitors (HMTi) and histone deacetylase inhibitors (HDACi)

Epigenetic dysregulation leads to many diseases including cancer. Cancer is a complex disease driven by genetic, epigenetic and environmental changes. Indeed, non-mutational epigenetic programming is one of the hallmarks of cancer. The epigenetic upregulation of oncogenes and downregulation of tumour suppressor genes are key processes in the development of cancer. DNMT, HDAC and HMT are dysregulated in several cancers, making them therapeutic targets for the treatment of cancer (Fig. 1**)** [12–15]. The U.S Food and Drug Administration (FDA) has approved the epigenetic drugs (epi-drugs); DNA methyltransferase inhibitors (DNMTi), histone deacetylase inhibitors (HDACi) and histone methyltransferase inhibitors (HMTi) for the treatment of several cancers including myelodysplastic syndromes, leukemia, lymphoma, multiple myeloma and epithelioid sarcoma [16–18]). The histone ‘reader’, bromodomain and extra-terminal motif (BET) protein that recognises acetylated lysine has additionally shown to be a promising target in cancer therapy. BET inhibitor (BETi) epi-drugs have demonstrated anti-cancer activity in both pre-clinical and clinical trials and function by binding reversibly to the bromodomain of BET proteins and subsequently disrupt vital histone-protein interactions [19, 20]. In this review, we focus on DNMT, HDAC and HMT, the epi-drugs DNMTi, HDACi and HMTi, and their clinical application as anti-cancer drugs in combination with other cancer therapies.

## DNMT, HDAC and HMT and the epigenetic drugs DNMTi, HDACi and HMTi

DNA methylation, which is a reversible covalent modification of DNA, is the most stable epigenetic modification, and associated with gene repression. In vertebrates, DNA methylation consists of the attachment of a methyl group to the C5 position of cytosine and occurs mainly in CpG dinucleotides (Fig. 1). Methylated cytosine represses transcription via inhibiting binding of transcription factors or by promoting the binding of transcriptional repressors. The DNMT family of enzymes are responsible for DNA methylation. This family consists of five members, DNMT1, DNMT2, DNM3a, DNMT3b and DNMT3L, where DNMT1, DNMT3a and DNMT3b methylate DNA, DNMT2 methylates RNA and while DNMT3L lacks catalytic activity, it, has been shown to regulate *de novo* DNA methylation [3, 21, 22]. Additionally, the ten-eleven translocation (TET) family of proteins have been shown to play an important role in DNA methylation, where the TET proteins oxidise 5-methylcytosines (5mCs) and lead to locus-specific reversal of DNA methylation. Furthermore, *TET* genes, and in particular *TET2* has been found to be frequently mutated in several cancers, notably hematological malignancies [23–25]. Aberrant DNA methylation is a hallmark of cancer cells, with both DNA hypermethylation and hypomethylation associated with oncogenesis [26, 27]. DNMTi can be classed into two main groups, nucleoside analogues and non-nucleoside analogues. DNMTi nucleoside analogues directly integrate into DNA or covalently bind to DNMT, are toxicity prone and include cytidine and S-adenosyl-L-homocysteine derivatives. Nucleoside DNMTi covalently bind to DNMT1, blocking DNMT function and leading to demethylation of CpG sites upon DNA replication. The resulting DNA-DNMT adducts induce DNA damage, resulting in the induction of the DNA damage response. A recent study demonstrated that the induction of DNA damage by DNMTi, in the presence of DNMT1, without the re-expression of tumour suppressors, was responsible for the anti-cancer effects of DNMTi [28–32]. At higher doses, nucleoside DNMTi can result in cytoxicity by incorporating into DNA or RNA and thus, the development of non-nucleoside DNMTi has gained interest [33]. Non-nucleoside DNMTi include predominantly natural products and small molecules, are not integrated into DNA and are less toxic, however, have lower efficacy and poorer selectivity than nucleoside DNMTi [31, 32, 34]. DNMTi are the most widely used epi-drugs in cancer therapy. The first FDA approved epi-drug was azacitidine (Vidaza; Celgene). Azacitidine and decitabine (5 aza 2´ deoxycytidine) (Dacogen; SuperGen) have FDA approval for the treatment of myelodysplastic syndromes (MDS), acute myeloid leukemia (AML) and chronic myelomonocytic leukemia (CMML). On May 20, 2022, the FDA granted accelerated approval for azacitidine for the treatment of patients with juvenile myelomonocytic leukemia (JMML) **(**Table 1**)** [35, 36]. In addition to the treatment of hematological malignancies, DNMTi may also have applicability in patients with solid tumours [37].

**Table 1:** DNA methyl transferase inhibitors, histone deacetylase inhibitors and histone methyl transferase inhibitors that are FDA approved for the treatment of cancer

Epigenetic drug	Target	FDA approved	Date of approval	Approved cancer indication
Azacitidine (Onureg, Vidaza)	DNA methyl transferase (DNMT)	Yes	2004	Acute myeloid leukemia (AML), juvenile myelomonocytic leukemia, myelodysplastic syndromes (MDS), chronic myelomonocytic leukemia (CMML) and juvenile myelomonocytic leukemia (JMML)
Decitabine (Dacogen)	DNMT	Yes	2006	AML, MDS and CMML
Vorinostat (SAHA)	Histone deacetylase (HDAC)	Yes	2006	Cutaneous T-cell lymphoma (CTCL)
Romidepsin (FK288)	HDAC	Yes	2009	CTCL
Belinostat (PXD101)	HDAC	Yes	2014	Peripheral T-cell lymphoma (PTCL)
Panobinostat (LBH589)	HDAC	Yes	2015	Multiple myeloma and CTCL
Tazemetostat	Histone methyl transferase (HMT)	Yes	2020	Epithelioid sarcoma and follicular lymphoma

Histone acetylation was first described in 1964 and is regulated by the opposing action of HAT which serve to add acetyl groups to lysine residues, and HDAC which remove acetyl groups (Fig. 1). Histone acetylation regulates transcription as acetylation leads to a relaxed chromatin confirmation which enables transcriptional activation, while HDAC are transcriptional repressors. There are 18 HDAC, which are classified into four classes (Class I, II, III and IV) depending on their homology to yeast HDAC [38–40]. Aberrant acetylation is associated with many cancers, with high expression of HDAC associated with poor prognosis in ovarian cancer, endometrial cancer, gastric cancer, neuroblastoma and multiple myeloma [41–45]. Furthemore, several studies have shown that HDAC are required for tumour growth and proliferation, cell cycle progression, cell differential, cell migration, drug resistance, autophagy, angionesis and apoptosis, further demonstrating the importance of HDAC as anti-cancer targets [46–48]. SAHA (Vorinostat; Merck & Co., Inc.) in 2006 was the first FDA approved HDACi for treating cutaneous T-cell lymphoma (CTCL) [49]. In 2009, the FDA approved FK228 (Romidepsin; Celgene) for the treatment of CTCL for patients who received at least one prior systemic therapy [50]. Belinostat (PXD101; Spectrum Pharmaceuticals, Inc) was FDA approved in 2014 for the treatment of peripheral T-cell lymphoma (PTCL) [51]. The most recent HDACi approved by the FDA was Panobinostat (Farydak; Novartis Pharmaceuticals) approved in 2015 for multiple myeloma [52]. A summary of the HDACi that are currently FDA approved are summarised in Table 1. In addition to their use in hematological malignancies, HDACi have also been investigated for the treatment of solid tumours [15, 53, 54]. However, HDACi have shown poor therapeutic efficacy as a monotherapy for the treatment of solid tumours [46]. In a study that aimed to understand the molecular basis of why HDACi respond poorly in solid tumours, the authors found that in breast cancer cells, the feedback activation of the leukemia inhibitory factor receptor (LIFR) signaling, limited the response to HDACi. HDACi was shown to increase histone acetylation of the *LIFR* gene promoter, which in turn facilitated BRD4-mediated transcriptional activation of LIFR. However, the upregulation of LIFR was found to activate JAK1-STAT3 signaling, which limited the response to HDACi. The authors show that the combination of HDACi with JAK1 or BRD4 inhibition sensitises breast cancer cells to HDACi, and suggest this combination treatment strategy for the treatment of breast cancer [55]. In a phase II clinical trial performed to examine the clinical and molecular response in lung cancer patients treated with the HDACi romidepsin, of the 19 patients receiving the HDACi, 9 patients showed a transient stabilisation of the disease. Romidepsin was found to increase H4 acetylation, increase p21 expression in lung tumour cells, without inducing significant cardiac toxicities, further warranting using romidepsin in combination with other cancer therapies [56]. Additional HDACi including valproic acid, tacedinaline, mocetinostat, abexinostat, entinostat, pracinostat and quisinostat are under clinical assessment for hematological and solid tumours [17, 57–63]. A recent study demonstrated that pracinostat significantly inhibited breast cancer metastasis and growth, through the inactivation of the IL-6/STAT3 signalling pathways. Furthermore, the authors found that pracinostat is a broad-spectrum pan-HDACi with better anti-metastatic properties than vorinostat [61]. HDACi may target a specific HDAC or be a pan-HDACi that targets all HDAC. HDACi bind to key Zn^2+^ ions of HDAC enzymes, inhibiting the catalytic function of HDAC, leading to an increase in histone acetylation. This results in an open chromatin confirmation allowing binding of DNA targeting agents, leading to the following anti-tumour effects including cell cycle arrest, tumour suppression, interfering with angiogenesis, inhibition of DNA repair, enhancing immune responses and inducing apoptosis [17, 41, 54, 64].

Histone methylation is a reversible histone modification catalysed by HMT which employ the cofactor S-5′-Adenosyl-l-Methionine as a methyl group donor to transfer a methyl group to predominantly lysine or arginine residues on histones H3 and H4 [65] (Fig. 1). In line with the amino acids that they modify, HMT belong to two families, histone lysine methyltransferases (KMT) or protein arginine methyltransferases (PRMT). KMT can be further subdivided into two families, SET domain-containing KMT and non-set domain containing KMT. SET domain-containing KMT include Su(var)3–9, Enhancer of Zeste (EZH) and Trithorax, while non-SET domain-containing KMT include the DOT1-like proteins. The PRMT family includes PRMT1-9 proteins. HMT plays vital roles in many cellular processes including transcriptional regulation, cell cycle progression, stress response, cell proliferation and differentiation [66]. Aberrant expression of HMT has been associated with kidney disease and different types of cancers [66–68]. Somatic mutations of *EZH2* which affect its activity have been identified in both solid and hematological malignancies. *EZH2* overexpression has been observed in several solid malignancies, where overexpression led to significantly higher gene repression, resulting in cancer growth, immunity and metastasis and poorer patient outcomes. *EZH2* was shown to be amplified in 10–15% of tumours proposing that it results in tumour formation. Furthermore ectopic expression of EZH2 was shown to shorten G1 of the cell cycle and confer a proliferative advantage to primary mouse embryonic fibroblasts, leading to the proposal that EZH2 is a *bona fide* oncogene [69–74]. In addition to the two FDA approved DNMTi ‘writers’ and four HDACi ‘erasers’, on June 18, 2020, Tazemetostat (TAZVERIK, Epizyme, Inc.), was added to the list of FDA approved epi-drugs. Tazemetostat, is a first-in-class inhibitor of the EZH2 HMT ‘writer’, used for the treatment of follicular lymphoma and epithelioid sarcoma [18, 75]. Currently, additional EZH2 inhibitors, valemetostat, CPI-1205 and PF-06821497, are under clinical development (Tables 1 and 2). A summary of the FDA approved epi-drugs for the treatment of cancer are discussed in Table 1.

**Table 2:** Clinical trials combining epigenetic drugs with immune checkpoint inhibitors

Epigenetic drug	Immune checkpoint inhibitor	Clinical trial ID	Cancer(s)	Status	Phase I/II/III clinical trial
Azacitidine	Pembrolizumab	NCT02546986	Non-small cell lung cancer (NSCLC)	Active	II
Azacitidine and entinostat	Nivolumab	NCT01928576	NSCLC	Completed	II
Azacitidine	Nivolumab	NCT03092674	Acute myeloid leukemia (AML) and myelodysplastic syndrome (MDS)	Active	II/III
Decitabine	Nivolumab	NCT05089370	Mucosal melanoma	Recruiting	I/II
Decitabine	Pembrolizumab	NCT03969446	AML and MDS	Recruiting	I
Decitabine	Ipilimumab	NCT02890329	AML and MDS	Active	I
Decitabine	PD-1 inhibitor	NCT05137886	Hodgkin’s lymphoma	Recruiting	II
Vorinostat	Pembrolizumab	NCT02638090	NSCLC	Active	I/II
Vorinostat	Pembrolizumab	NCT04357873	Mucosal cancers of head and neck, lung, cervix, anus, vulva and penis	Active	II
Vorinostat	Pembrolizumab	NCT03150329	Lymphoma	Active	I
Vorinostat	Pembrolizumab	NCT02619253	Renal, urothelial cell carcinoma and prostate	Completed	I
Romidepsin	Pembrolizumab	NCT03278782	T-cell lymphoma	Active	I/II
Romidepsin	Nivolumab	NCT02393794	Breast	Active	I/II
Panobinostat	Ipilimumab	NCT02032810	Melanoma	Completed	I
Panobinostat	Spartalizumab	NCT02890069	Colorectal, NSCLC, breast and renal	Completed	I
Tazemetostat	Durvalumab	NCT04705818	Solid tumours	Recruiting	II
Tazemetostat	Pembrolizumab	NCT03854474	Urothelial carcinoma	Active	I/II
Tazemetostat	Pembrolizumab	NCT05467748	NSCLC	Not yet recruiting	I/II
Tazemetostat	Pembrolizumab	NCT05353439	Small cell lung cancer	Recruiting	I
Tazemetostat	Pembrolizumab	NCT06242834	Non-Hodgkin’s lymphoma	Not yet recruiting	II
Valemetostat	Pembrolizumab	NCT05879484	Head and neck	Not yet recruiting	I
Valemetostat	Ipilimumab	NCT04388852	Prostate, urothelial and renal	Recruiting	I
Valemetostat	Atezolizumab and Bevacizumab	NCT06294548	Hepatocellular carcinoma	Not yet recruiting	I/II
CPI-1205	Ipilimumab	NCT03525795	Advanced solid tumours	Completed	I/II

NIH clinical trial database: www.clinicaltrials.gov

## Resistance to epigenetic therapies and rational for combination therapy

Resistance to current anti-cancer treatments including chemotherapy, targeted therapies and immunotherapy are inevitable [76, 77]. Similarly, resistance to DNMTi, HDACi and HMTi epigenetic therapies are often seen, which can lead to treatment failure and disease progression. To circumvent this problem, combination therapy, which reduces toxicity and drug resistance by administering lower drug doses, has been used as an approach to reinvigorate a therapeutics’ effectiveness against a particular cancer [15, 17, 78, 79].

A recent study demonstrated that *DNMT1* deletion led to resistance to DNMTi treatment in colon, breast and ovarian cells. The DNMTi decitabine, azacitidine and aza-T-dCyd were used in this *in vitro* and *in vivo* study, where all three DNMTi inhibited the expression of DNMT1, inhibited cell growth and induced cytotoxicity and/or apoptosis of colorectal cancer cells. Depletion of *DNMT1* was shown to markedly reduce the growth inhibition, cytoxicity and abrogated the cell cycle arrest, DNA damage and apoptosis in cancer cells. The authors concluded that patients with *DNMT1* deletions may not respond to DNMTi [80]. In a follow up study, the authors showed a link between TET2 and resistance to DNMTi in *DNMT1* depleted colorectal cancer cells. The study demonstrated that *DNMT1* deletion in colorectal cancer cells led to an upregulation of TET2 expression, re-expression of tumour suppressor proteins (p16^ink4A^ and p15^ink4B^), *CDKN2A* promoter demethylation, and associated resistance to DNMTi in *DNMT1*-deleted cells [81].

The mechanism of resistance to HDACi remains largely unknown. A study hypothesised that in human cells, the resistance seen to HDACi is an essential evolutionary consequence of the environmental exposure to HDACi. The authors speculated that cancers that were initially sensitive to HDACi, can developed mutations that alter how a cancer cell responds to HDACi. The authors further suggest that the identification of these mutations could aid the selective targeting of cancers sensitive to HDACi [82]. Two separate studies demonstrated that truncating mutations in *HDAC2* in human cancers with microsatellite instability led to a loss of HDAC2 protein expression and enzymatic activity and resulted in resistance to HDACi, highlighting the importance or determing *HDAC2* mutational status prior to treatment [83, 84]. Other studies have shown that elevated levels of Bcl-2, Trx, peroxiredoxins, p21, MAPK, PI3K, STAT3 and thioredoxin, constitute activation of NFκB through p65 acetylation and epigenetic and chromatin changes, alterations in the expression of HDAC and variations in drug efflux mechanisms, are involved in HDACi resistance mechanisms [17, 85, 86].

Mutations in the *SMARCB1* tumour suppressor gene led to the development of epithelioid and rhabdoid sarcomas, and loss of *SMARCB1* upregulates the expression of EZH2. Tazemetostat as an EZH2 inhibitor suppresses tumourigenesis in *SMARCB1*-deficient cells. However, not all patients respond to tazemetostat and in those who initially respond, drug resistance has been observed [87]. A recent publication demonstrated the molecular mechanism of tazemetostat resistance in *SMARCB1*-deficient tumours. The authors identified distinct acquired mutations which converges on the RB1/E2F axis and result in decoupling of EZH2-dependent differentiation and cell cycle control, allowing tumour cells to escape tazemetostat-induced G1 arrest. The authors suggest a rational epigenetic combination therapy strategy to overcome the clinical resistance to tazemetostat in epithelioid sarcomas, rhabdoid tumours and other epigenetically dysregulated cancers [88]. Thus, combining epi-drugs with other cancer therapies may provide a mechanism to achieve their full therapeutic potential. The following sections of this review will focus on the combination of epi-drugs with other cancer therapies.

### Combination of epigenetic therapies with immunotherapy

Immune checkpoint inhibitors (ICI) have revolutionised the cancer treatment landscape for solid tumours and haematological malignancies. ICI keep T-cells active to fight tumour cells. The immune checkpoint proteins, which include cytotoxic T lymphocyte-associated antigen 4 (CTLA-4) and programmed cell death receptor 1 (PD-1), and their respective ligands, CD80/CD86 and PD-L1, are the most in-depth studied. In ICI therapy, monoclonal antibodies against PD-1/PD-L1 and CTLA-4/CD80/86, are used to reverse the inhibitory effects of immune checkpoint proteins and enable anti-tumour responses in particular immune privilege [89, 90].

Ipilimumab which blocks the checkpoint protein CTLA-4, was the first FDA approved ICI in 2011, for the treatment of malignant melanoma [91, 92]. Prembrolizumab (Keytruta) was the first humanised monoclonal anti-PD-1 antibody, FDA approved in 2014 for the treatment of unresectable or metastatic melanoma [93] and since, expanded to treat many other cancers [94]. An additional five ICI have been FDA approved, and include two PD-1 inhibitors (nivolumab and cemiplimab) and three PD-L1 inhibitors (atezolizumab, avelumab and durvalumab) to treat approximately 15 different types of cancers [95]. Several new immune checkpoints targets and new ICI are under clinical assessment [96]. The use of prognostic and predictive biomarkers is necessary to best select patients who will respond to the ICI therapy. At present, PD-L1 expression is the only predictive biomarker to predict a response to ICI therapy and has its limitations. Responses to ICI are observed in tumours that do not express PD-L1 in 6.5 to 10% of cases, and expression of PD-L1 doesn’t provide a certainty of treatment response or treatment resistance [97]. A study that examined the analysis of FDA approvals of ICI demonstrated that in less than 30% of studies did PD-1 positivity predict increased response, and moreover, only 20% of approvals included companion PD-L1 diagnostic testing, despite over 80% of trials that led to FDA approval showed PD-L1 correlation [98]. Furthermore, other factors have been shown to influence PD-L1 expresion such as tumour heterogeneity, the antibody and methodology used for staining, variability in timing of tissue collection, the role of PD-L1 on tumour infilteratng lymphocytes and other immune cells located in the immediate vicinity [97, 99]. Other predictive and prognostic biomarkers that include the tumour mutation burden, microbiome, microsatellite instability, interferon-gamma, extracellular matrix, hypoxia and radiomics, have additionally been described, however, some require further validation [97].

As with other cancer therapies, most patients show primary or acquired resistance to ICI. The immune phenotype of the tumour microenvironment (TME), the tumour mutational burden and tumour immune evasion, remain challenges that limit the success of ICI. Thus, combination of ICI with other cancer therapeutics has emerged as strategy to overcome this resistance. Epi-drugs have been shown to enhance anti-tumour immune responses. The combination of epi-drugs with ICI can synergistically enhance the immune response by modulating gene expression and by reversing the immune suppression in the TME [100–102]. While this field of study has only recently emerged, there are multiple studies highlighting their potential combination. A phase Ib clinical trial of vorinostat with pembrolizumab in metastatic renal, urothelial and prostate cancer patients was performed to determine the safety and tolerability of the combination therapy. The study concluded that vorinostat and pembrolizumab combination was relatively well tolerated and is additionally active in a subset of IC-resistant urothelial and renal cancer patients and prostate cancer patients who are IC-naïve (ClinicalTrial.gov identifier: NCT02619253). A phase II randomised study of epigenetic priming with azacitidine and entinostat followed by the ICI, nivolumab, compared to nivolumab alone in NSCLC patients, was recently completed (ClinicalTrial.gov identifier: NCT01928576). This study demonstrated that sequential therapy with epigenetic priming pre-ICI was safe and was subsequently expanded to include patients who had previously received ICI and concurrent epigenetic therapy with nivolumab. Although limited clinical efficacy was observed, the study was able to identify patients who benefited from the combination therapy and furthermore molecular analysis demonstrated the effect of epigenetic priming on re-shaping the TME. A summary of some epi-drugs in combination with ICI in clinical trials for the treatment of cancer, is discussed in Table 2.

Epigenetic changes within immune checkpoint genes may contribute towards resistance to ICI and these modifications may be a predictive biomarker to ICI therapy or may be targets in combination therapies. A study demonstrated that increased expression of PD-L1, post-azacitidine treatment, elevated the anti-PD1 therapy response, while in melanoma, lower *CTLA-4* methylation resulted in a better response to anti-CTLA-4 or PD1 therapy [103, 104].

### Combination of epigenetic therapies with DNA repair inhibitors or radiotherapy

DNA in our cells is under constant threat from both exogenous and endogenous sources, and if unrepaired, may result in genomic instability or malignant transformation. The DNA damage response (DDR) responds to these threats and functions to repair this damage and maintain genomic stability [105, 106]. DNA double strand breaks (DSBs) include the most damaging lesions to cells. DSBs are repaired by two main mechanisms, non-homologous end-joining (NHEJ) and homologous recombination (HR). While NHEJ re-joins the broken ends without using extensive homology, HR requires a sister chromatin and can thus only be used during certain phases of the cell cycle [107].

In cancers, several DDR genes are epigenetically regulated, resulting in transcriptional silencing and a downregulation in the DNA repair capacity of the tumour, which may result in chemosensitivity to DNA damaging therapeutics. Furthermore, in cancer cells, the upregulation of the DDR has been suggested to lead to resistance to genotoxic therapies and hence, inhibiting the DDR can lead to better treatment outcomes [108, 109]. HDACi have been shown to cause DNA damage that normal and not cancer cells can repair. A study demonstrated that vorinostat induced DSBs in LNCaP (prostate cancer cells) and A549 (lung cancer cells) cancer cells which were unable to be repaired by these cells, while the normal cells (HFS) could repair this damage. Vorinostat was additionally found to suppress the DNA repair proteins, RAD50 and MRE11, in cancer cells and not normal cells, collectively resulting in cancer cell death [110]. The advent of PARP inhibitors has considerably reinvigorated the interest of DNA repair inhibitors in cancer therapy. Olaparib (AZD-2281, Lynparza), an inhibitor of the DNA repair protein poly ADP ribose polymerase (PARP), was initially FDA approved in 2014 for the treatment of germline BRCA-mutated advanced ovarian cancer patients who had received prior chemotherapy [111]. For BRCA-proficient cancers, PARP inhibition fails as a single agent and therefore combination therapy may expand its use. Currently, there are several on-going studies [112–114] and clinical trials combining epi-drugs with olaparib (Table 3). A study demonstrated that in BRCA-proficient non-small cell lung cancer cells, PARP inhibition synergises with DNMTi in reprogramming the DNA repair transcriptome and creating a HR defect, which sensitises the cells to PARP inhibition. Furthermore, NHEJ was also found to be downregulated, leading to ionizing radiation sensitivity [112]. Additionally, the use of the DNMTi, decitabine and azacitidine, in combination with PARP inhibition was investigated in a pre-clinical study for the treatment of acute myeloid leukemia and triple negative breast cancer. The authors demonstrated that the combination therapy was a compelling strategy for treating these cancers, independent of BRCA mutation [113]. More recently, the combination of olaparib and the DNMTi, azacitidine, was assessed in epithelial ovarian cancer cells using *in vitro* and *in vivo* analyses. The authors found that the combination strategy had a significant anti-tumour effect in the epithelial ovarian cancer cells, suggesting the potential use of this combination therapy for the treatment of epithelial ovarian cancer [114]. There are several other promising cancer therapeutics targeting DNA repair pathways under development [34, 115–118], which may have even greater effects when used in combination with epi-drugs. A summary of some epi-drugs in combination with DNA repair inhibitors for the treatment of cancer, is discussed in Table 3.

**Table 3:** Clinical trials combining epigenetic drugs with DNA repair inhibitors or radiotherapy

Epigenetic drug	Combination treatment	Clinical trial ID	Cancer(s)	Status	Phase of clinical trial
Decitabine	DNA repair inhibitor (Olaparib)	NCT06177171	Advanced solid tumours	Recruiting	I
Vorinostat	Olaparib	NCT03742245	Breast	Recruiting	I
Vorinostat	Olaparib	NCT03924245	Refractory lymphomas	Active, not recruiting	I
Decitabine	Radiotherapy	NCT03445858	Solid tumours and lymphoma	Active, not recruiting	I
Decitabine	Radiotherapy	NCT01707004	Acute myeloid leukemia	Completed	II
Vorinostat	Radiotherapy	NCT00821951	Non-small cell lung cancer	Completed	I
Vorinostat	Radiotherapy	NCT00838929	Brain	Completed	I
Vorinostat	Radiotherapy	NCT00731731	Glioblastoma	Completed	I/II
Vorinostat	Radiotherapy	NCT02420613	Brain	Active, not recruiting	I
Vorinostat	Radiotherapy	NCT05608369	Head and neck squamous cell carcinoma	Not yet recruiting	II
Belinostat	Radiotherapy	NCT02137759	Glioblastoma multiforme	Active, not recruiting	I
Panobinostat	Radiotherapy	NCT00670553	Prostate, esophageal and head and neck	Completed	I
Panobinostat	Radiotherapy	NCT05009992	Glioma	Recruiting	II
Tazemetostat	Radiotherapy	NCT05151588	Sinonasal carcinoma	Not yet recruiting	II

NIH clinical trial database: www.clinicaltrials.gov

Radiotherapy which remains as one of the most extensively utilised cancer treatments causes cell death by inducing mainly DSBs [119]. Several studies have demonstrated that the combination of epi-drugs with radiotherapy results in increased sensitivity to radiotherapy by upregulating oxidative stress, arresting the cell cycle and preventing DNA repair [120]. An *in vivo* study demonstrated that vorinostat, in combination with radiotherapy, improved the objective response rate in refractory neuroblastoma. This study demonstrated that in neuroblastoma, vorinostat potentiates the anti-neoplastic effects of irradiation by down-regulating the DNA repair protein, Ku-86 [121]. Vorinostat in combination with radiotherapy and capecitabin was shown to significantly increase the overall survival of pancreatic ductal adenocarcinoma patients in a phase I clinical trial [122]. A phase I clinical study also demonstrated that vorinostat in combination with radiotherapy could improve the objective response rate of gastrointestinal carcinoma [123]. A summary of some epi-drugs in combination with radiotherapy for the treatment of cancer, is discussed in Table 3.

### Combination of epigenetic therapies with platinum-based chemotherapeutics

In 1978 the FDA approved cisplatin (*cis*-diamminedichloroplatinum (II)) for the treatment of testicular and bladder cancer [124]. Since then, cisplatin has been used as a first-line treatment (either as a monotherapy or combination therapy) for several solid malignancies including ovarian, bladder, head and neck, testicular, lung, colorectal and cervical cancers. Cisplatin acts through the generation of DNA damage in the form of DNA adducts, activation of the DDR, followed by apoptosis. However, cisplatin is associated with severe side effects, including nephrotoxicity, neurotoxicity and ototoxicity and chemoresistance [125, 126]. Second-generation platinum compounds were developed in the 1980s in the hope of reducing the severe side effects associated with cisplatin. Carboplatin (*cis*-diamine (cyclobutene-1,1-dicarboxylate-*O*,*O´*) platinum (II)) is an analogue of cisplatin and was FDA approved for the treatment of ovarian cancer in 1989. Carboplatin is associated with less nephrotoxicity and neurotoxicity than cisplatin, however, generates the same DNA adducts. However, most cisplatin-resistant tumours are also carboplatin-resistant [126, 127]. This led to the development of the third generation of platinum drugs, oxaliplatin ([(IR,2R)-cyclohexane-1,2-diamine](ethanedioato-*O*,*O´*) platinum (II)), which has distinct immunological and pharmacological properties compared to carboplatin and cisplatin and is clinically used to treat several cancers. Nevertheless, some cross-resistance in the clinic between oxaliplatin and cisplatin has been observed [126, 128, 129].

Although an initial treatment response is observed with platinum-based chemotherapeutics, in most cases, chemoresistance is eventually observed, leading to treatment failure [126]. It has been shown that the DDR genes *BRCA1/2* regulate chromatin confirmation and enhancer activity. These studies suggest that the combination of epi-drugs with chemotherapy may enhance chemotherapy outcomes by circumventing acquired resistance and through chromatin decompaction, epi-drugs may additionally improve the accessibility of chromatin to chemotherapeutics [130, 131]. A study combining vorinostat and low dose cisplatin on oral squamous cell carcinoma cells demonstrated that compared to each individual treatment, the combination treatment synergistically induced cytoxicity and apoptosis of the cells. The authors suggested that the concurrent treatment of cisplatin and vorinostat enhanced the tumour cells’ sensitivity to subtoxic doses of cisplatin [132]. Furthermore, a recent study demonstrated that the sequential treatment of the DNMTi, azacitidine, and carboplatin induced immune activation of platinum-resistance ovarian cancer cells lines and primed the ovarian cells to be more responsive to ICI therapy [133]. A summary of some epi-drugs in combination with platinum-based chemotherapeutics in ongoing or completed clinical trials, for the treatment of cancer are discussed in Table 4.

**Table 4:** Clinical trials combining epigenetic drugs with platinum-based drugs

Epigenetic drug	Platinum drug	Clinical trial ID	Cancer(s)	Status	Phase I/II/III clinical trial
Azacitidine and valproic acid	Carboplatin	NCT00529022	Ovarian	Completed	I
Azacitidine, epirubicin and capecitabine	Oxaliplatin	NCT01386346	Esophageal	Completed	I
Decitabine	Carboplatin	NCT05983276	Ovarian	Recruiting	II
Decitabine	Carboplatin	NCT02957968	Breast	Active	II
Decitabine	Carboplatin	NCT00477386	Ovarian	Completed	I/II
Decitabine	Oxaliplatin	NCT04049344	Advanced renal cell carcinoma	Unknown	II
Vorinostat	Carboplatin	NCT01281176	Advanced solid tumours	Active	I
Vorinostat	Cisplatin	NCT05608369	Head and neck squamous cell carcinoma	Not yet recruiting	II
Vorinostat	Carboplatin	NCT00287937	Solid tumours	Completed	I
Vorinostat	Carboplatin	NCT00481078	Non-small cell lung cancer (NSCLC)	Completed	II
Romidepsin	Carboplatin,	NCT01590732	T-cell lymphoma	Completed	I
Romidepsin	Cisplatin	NCT02393794	Breast	Active	I/II
Belinostat	Carboplatin	NCT00993616	Ovarian, fallopian tube and peritoneal	Completed	II
Belinostat	Carboplatin	NCT01310244	NSCLC	Completed	I/II
Belinostat	Cisplatin	NCT06406465	Neuroendocrine carcinomas	Not yet recruiting	II
Panobinostat	Carboplatin	NCT00556088	Solid tumours	Completed	I
Panobinostat	Carboplatin	NCT01169636	Hodgkin lymphoma	Completed	I/II
Panobinostat	Cisplatin	NCT01336842	Solid tumours	Completed	I
Tazemetostat	Cisplatin	NCT05151588	Sinonasal carcinoma	Not yet recruiting	II

NIH clinical trial database: www.clinicaltrials.gov

### Combination of epi-drugs DNMTi, HDACi and HMTi

As DNA methylation and histone post-translational modifications operate in parallel, there is an ongoing interest in combining epigenetic therapies, as this may lead to an increase in the efficacy of each individual treatment [134]. A study that demonstrated that the re-expression of methylated genes silenced in cancer, occurred after the combination of a DNMTi followed by HDACi, led to a substantial interest in the combination of epi-drugs [135]. There are several ongoing and completed clinical trials of combinations of DNMTi, HDACi and HMTi, for the treatment of cancer (Table 5). A phase II multicentre study of oral azacitidine in combination with romidepsin in patients with peripheral T-cell lymphoma demonstrated that the combination treatment induced a high response rate and increased the remission of these patients (ClinicalTrial.gov identifier: NCT01998035) [136]. Another study of low-dose adjuvant azacitidine in combination with entinostat was shown to disturb the premetastatic microenvironment and inhibit the growth and formation of lung metastasis [137]. Furthermore, a recent study combining the HDACi, belinostat, with the HMTi, tazemetostat, promoted antigen presentation pathways in germinal cancer-diffuse large B-cell lymphoma (GC-DLBCL) cells. This study demonstrated that the combination therapy could restore immunogenicity in GC-DLBCL and improve the immune-mediated killing of the tumour cells [138]. There is an ongoing clinical phase I/expansion clinical trial (ClinicalTrial.gov identifier: NCT05627245) Table 5, combining tazemetostat and belinostat for the treatment of relapsed or refractory lymphoma. The primary outcome of this study is to determine the maximum tolerated dose. The secondary outcomes of this study include overall response, disease response, overall survival, progression free survival, the total number of cycles, duration of response, number of dose delays, number of dose reductions, adverse events and pharmacokinetics.

**Table 5:** Clinical trials combining epigenetic drugs

Epigenetic drug	Epigenetic drug	Clinical trial ID	Cancer(s)	Status	Phase I/II/III clinical trial
Azacitidine	Vorinostat	NCT00392353	Acute myeloid leukemia (AML) and myelodysplastic syndrome (MDS)	Active	I/II
Azacitidine	Vorinostat	NCT00336063	Nasopharyngeal and T-cell lymphoma	Active	I
Azacitidine	Vorinostat	NCT03843528	Childhood myeloid malignancies	Recruiting	I
Azacitidine	Vorinostat	NCT01522976	MDS and leukemia	Active	II
Azacitidine	Vorinostat	NCT01983969	Lymphoma	Completed	I/II
Decitabine	Vorinostat	NCT00357708	Hematologic	Completed	I
Decitabine	Vorinostat	NCT00275080	Solid tumours, non-Hodgkin’s lymphoma and leukemia	Completed	I
Azacitidine	Romidepsin	NCT03703375	T-cell lymphoma	Active	III
Azacitidine	Romidepsin and belinostat	NCT04747236	Peripheral T-cell lymphoma	Recruiting	II
Azacitidine	Romidepsin	NCT03593018	T-cell Lymphoma	Active	III
Azacitidine	Romidepsin	NCT04447027	T-cell malignancies	Active	I
Decitabine	Romidepsin	NCT00114257	Leukemia and MDS	Completed	I
Decitabine	Romidepsin	NCT00037817	Pulmonary and pleural malignancies	Completed	I
Azacitidine	Belinostat	NCT00351975	Hematologic cancers	Completed	I
Decitabine	Belinostat	NCT04340843	Chondrosarcoma	Active	II
Azacitidine	Panobinostat	NCT01613976	MDS and leukemia	Completed	I
Azacitidine	Panobinostat	NCT00946647	MDS and leukemia	Completed	I/II
Decitabine	Panobinostat	NCT00691938	AML and MDS	Completed	I/II
Tazemetostat	Belinostat	NCT05627245	Lymphomas	Recruiting	I

NIH clinical trial database: www.clinicaltrials.gov

## Side effects, biomarkers and improving the efficacy of epigenetic therapies

Similar to other cancer therapeutics, epi-drugs are associated with side effects. The most common side effects for DNMTi include gastrointestinal symptoms, bleeding, anaemia, neutropenia, joint pain, bone marrow suppression and cytoxicity [139]. HDACi have been shown to cause a variety of side effects including thrombocytopenia, nausea, vomiting, anorexia, neutropenia, fatigue, diarrhoea and cardiac toxicity [17]. Six deaths were reported in patients treated with romidepsin prior to FDA approval [140, 141]. It is of importance to note that several clinical trials in combination with epi-drugs have been terminated due to slow accrual, low enrolment or unacceptable toxicity (ClinicalTrial.gov identifier: NCT04190056, NCT02395627, NCT01413750, NCT02900560, NT00702572, NCT00910000, NCT00662311, NCT00702962, NCT00976183, NCT00443261, NCT01324635).

The use of biomarkers to select patients or predict their response to epi-drugs will lower the side effects observed with this type of therapy. An *in vivo* study demonstrated that the upregulation of ERα and the downregulation of pAKT, HER2 and pHER2 was associated with a response to the HDACi entinostat [142, 143]. Furthermore, studies have shown that the expression of DCK, DNMT levels, DNA methylation, genomic assays and histone hyperacetylation are potential biomarkers in predicting the response to epigenetic therapies [142].

A recent study surprisingly showed that the pan-HDACi, vorinostat and LBH589, promoted breast cancer metastasis by elevating NEDD9 expression, both *in vitro* and *in vivo* [144]. Taken together with similar studies, these data strong suggest that HDACi should not be used as a monotherapy in breast cancer. These studies further highlight the importance of using biomarkers to select patients or predict their response to these epi-drugs in order to limit their adverse effects and exclude patients unlikely to benefit from these therapies.

The use of multi-target epi-drugs are currently being evaluated in clinic trials to improve the efficacy of epi-drugs. CUDC-101 was the first multitarget epi-drug to be trialled (ClinicalTrial.gov identifier: NCT01171924), which targets HDAC, epidermal growth factor receptor (EGFR) and human epidermal growth factor receptor 2 (HER2), in patients with advanced head and neck, breast, gastric, liver and lung cancers. CUDC-101 simultaneously antagonises different oncogenic targets and may additionally improve the treatment of heterogeneous and drug-resistant tumours, and thus provides several advantages over single-target cancer drugs. Currently, other multi-target epi-drugs (fimepinostat, tinostamustine and domatinostat) are under clinical assessment for the treatment of cancers (ClinicalTrial.gov identifier: NCT03893487, NCT03452930, NCT04133948) [134].

## Novel delivery methods of epi-drugs

Numerous delivery systems are currently being investigated to improve the specificity and efficacy of epi-drugs. The delivery of epi-drugs to their targets are challenged with poor solubility, poor permeability and poor pharmacokinetic properties such as a short half-life, quick metabolism and clearance. Novel delivery methods of epi-drugs include, prodrugs, chemical conjuagation, nanoparticle-based delivery and HDAC proteolysis targeting chimeras. The prodrug approach consists of conjugating a prodrug which is an inactive, biodegradable derivative of an epigenetic drug, which, is metabolised to a pharmacologically active drug upon uptake. The aim of a prodrug is to improve the ADMET (Absorption, Distribution, Metabolism, Excretion and Toxicity) of the epigenetic drug that it is conjugated to. At present, there are several HDACi prodrugs under development [134, 145]. While the epi-drugs azacitidine and decitabine are used for the treatment of cancer, it is difficult to achieve stable pharmacokinetics with these drugs, due to rapid deamination *in vivo* by cytidine deaminases and by spontaneous hydrolytic cleavage. A prodrug approach was taken to develop metabolically stable prodrugs of azacitidine, decitabine and additional new DNMTi. In this study, the authors synthesised 35 5´-O-trialkylsilylated azactidines/decitabines which were potentially resistance to deamination. Of these drugs, 11 exhibited similar demethylating activity and suitable aqueous solubility. *In vivo* pharmacokinetic analysis demonstrated that two prodrugs of decitabine showed greater metabolic stability, reduced toxicity and comparable DNA demethylation activity, further highlighting the significance of a prodrug approach [146]. Another approach is the chemical conjugation of molecules to epi-drugs, to improve upon their anti-cancer properties. The platinum-SAHA conjugate, *cis*-[Pt^II^(NH_3_)_2_(malSAHA_–2H_), was developed to be able to bind DNA, in a similar manner to platinum, and additionally lead to HDACi anti-cancer activity. An *in vitro* study demonstrated that *cis*-[Pt^II^(NH_3_)_2_(malSAHA_–2H_) was slightly less toxic in cells compared to cisplatin, with SAHA accumulating better in cancer cells. However, *cis*-[Pt^II^(NH_3_)_2_(malSAHA_–2H_) didn’t exhibit inhibition of HDACi activity, and was found to bind to DNA less readily than cisplatin. The malonate linker in the conjugate was found to be the reason for the reduced HDACi activity and future work to enhance DNA binding and HDACi activity is warranted [147]. When the platinum (IV) prodrug, diaminedichlorodihydroxyplatinum (ACHP), was conjugated to valproic acid, the authors observed synergistic cytotoxicity against many different cancer cell lines. Platinum (IV) is reduced to platinum (II) and valproic acid is released, which results in cell cycle arrest at S phase and induction of apoptosis. The conjugate drug additionally showed strong antiproliferative activity and low systemic toxicity in a lung cancer *in vivo* model [148]. Recent advances with nanoparticle-based therapeutics are being utilised to improve the targeted delivery of epi-drugs to their targets. The nanoparticle delivery mechanisms for epi-drugs include liposomes, polymer-based nanoparticles, micelles, dendrimers and inorganic nanoparticles [134, 149]. An *in vitro* study demonstrated that pegylated liposomes loaded with the HDACi trichostatin A, CG1521 and PXD101 demonstrated enhanced efficacy of the HDACi in breast cancer cell lines [150]. Furthermore, an *in vivo* study in rats with iron complexation to the HDACi, SAHA and LAQ824, in pegylated liposomes improved the overall aqueous solubility, *in vitro* release and pharmacokinetic properties of the HDACi [151]. A more recent study demonstrated that encapsulation of the HDACi, CG-1521, in a polymeric starch nanoparticle, did not interfere with its mechanism of action. Encapsulated CG-1521 resulted in cell cycle arrest and induction of apoptosis of breast cancer cells. Furthermore, encapsulation improved the therapeutic efficacy of CG-1521 by enabling a steady sustained release of CG-1521 and increasing its bioavailability and half-life [152]. Additionally, a study showed that when the HDACi, SAHA, was loaded in PEG-PCL polymeric micelles, the nanoformulation induced a better cell cycle arrest in G2/M, led to greater apoptosis and demonstrated significant tumour suppression *in vivo* compared to SAHA alone. Another study using PEG-PGLA copolymeric micelles for the delivery of SAHA demonstrated that in solid tumours, the nanoformulation improved the biopharmaceutical properties of SAHA and led to an enhanced anti-tumour efficacy [153, 154]. Another exciting upcoming field is HDAC proteolysis targeting chimeras (PROTAC) which are an alternative drug delivery technology to degrade proteins using the ubiquitin proteasome system. The integration of HDAC binding motifs in PROTAC results in chemical, targeted HDAC degradation. A recent study demonstrated that HDACI/II degradation by HDAC PROTAC leads to increased global gene expression and apoptosis, which is vital for the development of more effective HDACi [155, 156]. Taken together, these studies demonstrate the potential of combining epi-drugs with novel delivery methods to increase their specificity and anti-tumour efficacy.

## Conclusion

Over the last few years, we have witnessed a considerable expansion in our understanding of epigenetics and their subsequent application in cancer therapy. As epigenetic modifications in cancers are reversible, they are attractive targets for cancer therapy. Several epi-drugs are FDA approved for the treatment of cancer and include DNMTi, HDACi and HMTi. At present, there are two DNMTi, four HDACi and one HMTi epi-drugs that are FDA approved for the treatment of cancer, with several other inhibitors currently being tested in clinical trials [20].

A main drawback of epi-drugs are their less promising results in solid tumours, compared with their success in treating hematological malignancies. The use of novel delivery methods of epi-drugs including prodrugs, conjugation, nanoparticle-based delivery methods and PROTACS may address this issue, enabling a more effective intra tumour delivery of these drugs [149]. Furthermore, the use of multi-target epi-drugs in the future, may provide advantages over single-target cancer therapies by treating difficult to treat heterogeneous and drug-resistant tumours.

Nucleoside DNMTi are characterised by poor selectivity, high toxicity and low bioavailability, while non-nucleoside DNMTi have low efficacy and poor selectivity, which have limited the clinical applications of DNMTi. Identification of the structures of DNMT and inhibitor complexes and enrichment of DNMT chemical space may lead to the development of more selective, more effective and less toxic DNMTi [32]. While HDACi have been widely used in the clinic, their poor pharmacokinetics, limited specificity, low solubility, low permeability, toxic effects and the development of tumour resistance have limited their use. Combining HDACi with novel delivery methods may increase their specificity and anti-tumour efficacy, and thus their applicability in the clinic.

While immunotherapy has revolutionised the cancer treatment landscape for solid tumours and haematological malignancies, most patients show primary or acquired resistance to this treatment. Thus, combination with other cancer therapeutics has emerged as a recent strategy to overcome this resistance, and epi-drugs have been shown to enhance anti-tumour immune responses. This combination strategy has shown synergy in the clinical setting and is a novel attractive approach in the treatment of cancer [102]. Nonetheless, adverse events and off-target effects associated with the combination of ICI and epi-drugs need to be addressed in order to achieve their full therapeutic benefit.

With continued advances in AI and multi-omics, we will continue to see the development of novel tumour epigenetic biomarkers for screening, diagnosis and for the development of personalised cancer treatments. The identification of predictive biomarkers to select for patients or predict their response to epi-drugs requires extensive tissue-specific analysis, promoting personalised treatments, which will lower the associated side effects and improve patient outcomes. Taken together, epi-drugs are emerging as successful stand-alone or combination anti-cancer agents that may be key players in the future of personalised cancer therapy.

## Data Availability

No datasets were generated or analysed during the current study.

## Abbreviations

*Epi-drug*: Epigenetic drug
*DNMT*: DNA methyltransferases
*HAT*: Histone acetyltransferases
*HDAC*: Histone deacetylases
*HMT*: Histone methyltransferases
*FDA*: U.S Food and Drug Administration
*DNMTi*: DNA methyltransferase inhibitors
*HDACi*: Histone deacetylase inhibitors
*HMTi*: Histone methyltransferase inhibitors
*BETi*: BET inhibitors
*TET*: Ten-eleven translocation
*CTCL*: Cutaneous T-cell lymphoma
*PTCL*: Peripheral T-cell lymphoma
*AML*: Acute myeloid leukemia
*MDS*: Myelodysplastic syndromes
*CMML*: Chronic myelomonocytic leukemia
*JMML*: Juvenile myelomonocytic leukemia
*NSCLC*: Non-small cell lung cancer
*KMT*: Histone lysine methyltransferases
*PRMT*: Protein arginine methyltransferases
*ICI*: Immune checkpoint inhibitors
*CTLA-4*: Cytotoxic T lymphocyte-associated antigen 4
*PD-1*: Programmed cell death receptor 1
*TME*: Tumour microenvironment
*DDR*: DNA damage response
*DSBs*: DNA double strand breaks
*HR*: Homologous recombination
*NHEJ*: Non-homologous end-joining
*PARP*: Poly ADP ribose polymerase
*GC-DLBCL*: Germinal cancer-diffuse large B-cell lymphoma
*ADMET*: Absorption Distribution, Metabolism, Excretion, Toxicity
*EGFR*: Epidermal growth factor receptor
*HER2*: Human epidermal growth factor receptor 2
*PROTAC*: Proteolysis targeting chimeras
